# Development and psychometric properties of the human papillomavirus-quality of life (HPV-QoL) questionnaire to assess the impact of HPV on women health-related-quality-of-life

**DOI:** 10.1007/s00404-022-06583-4

**Published:** 2022-05-13

**Authors:** Pluvio J. Coronado, Carmen González-Granados, Mar Ramírez-Mena, Javier Calvo, María Fasero, Mónica Bellón, Javier F. García-Santos, Javier Rejas-Gutiérrez

**Affiliations:** 1 Women’s Health Institute, Hospital Clínico San Carlos, Instituto de Salud de La Mujer IdISSC, Universidad Complutense, C/Martín Lagos S/N, 28040 Madrid, Spain; 2 Department of Obstetrics and Gynaecology, Hospital Quirón, Málaga, Spain; 3 Department of Obstetrics and Gynaecology, Hospital Sanitas la Zarzuela, Universidad Francisco de Vitoria, Madrid, Spain; 4 EACCOS Research Group, Universidad Autónoma de Madrid, Madrid, Spain

**Keywords:** Human papillomavirus (HPV) infection, Women health-related quality-of-life, Development, HPV-QoL questionnaire

## Abstract

**Purpose:**

The HPV-Quality-of-Life (HPV-QoL) questionnaire was developed to determine the impact of Human-Papillomavirus (HPV) infection and related interventions on women health-related quality-of-life. This study provides the development and preliminary psychometric properties of a novel HPV-QoL questionnaire for adult women with HPV.

**Methods:**

After reviewing literature and cognitive debriefing interviews in women who had experienced HPV-related conditions, instrument items and domains were developed. A draft questionnaire was pilot tested for comprehension and ease of completion. Psychometric evaluation of the final HPV-QoL scale was conducted in a psychometric study including 252 adult women derived to our centre by a positive HPV test in the cervical cancer screening program and/or presenting genital warts.

**Results:**

The present study reveals that the HPV-QoL questionnaire, structured in four domains: general well-being [including psychological well-being and social well-being subdomains], health, contagiousness and sexuality, showed good metric properties of feasibility irrespective of age or educational level, and time to administer was less than 5 min. Internal consistency and temporal stability (reliability) showed values above the acceptable standards. The instrument showed its concurrent validity by means of a significant correlation with mental and sexual existing instruments; GHQ-12 and FSFI questionnaires, respectively, and also known groups validity showing significant differences among the subgroups regarding either sexual dysfunction or mental deterioration.

**Conclusion:**

This study provides an HPV-QoL questionnaire with an innovative patient-reported outcomes specific measurement tool to assess HRQoL in women with HPV infection. The present study suggests this questionnaire has satisfactory psychometric properties, including validity and reliability. Results support the use of the HPV-QoL questionnaire as a HRQoL measurement instrument for daily medical practice and clinical research.

## Introduction

Over the last decades, healthcare researchers have witnessed the breakthrough changes that have led to new concepts regarding the evaluation and appraisal of health care. Standard measures of morbidity and mortality, just evaluate the effectiveness of medical interventions in chronic diseases. When treatment of this diseases does not significantly modify survival rates and when pharmaceutical or other interventions may cause serious adverse events, there is a need to evaluate the effectiveness in a new way [1]. Health Outcomes Research (HOR) is a relatively recent discipline that focuses on the measurement of the impact of the disease and treatment upon patient perceived health, providing an answer to these new current medical concerns [2]. HOR is applied to clinical and population-based research, leading to the enhancement of the healthcare end results in terms of benefits to the patient and society.

Human papillomavirus (HPV) causes one of the most common sexually transmitted infections (STIs), and high-risk genotypes are related to cancer in several localizations [3, 4]. Diagnosed in more than 90% of cervical cancers, the fourth deadliest cancer in women, human papillomavirus (HPV) is currently the most common pathogen responsible for female cancers. Moreover, HPV infection is associated with many other diseases, including cutaneous and anogenital warts, and genital and upper aero digestive tract cancers [5–7]. Recently, features of the cervicovaginal microbiota are found to be associated with the incidence of HPV-related diseases, presenting a novel approach to identify high-risk women through both blood and cervical samples [5]. HPV infection usually occurs soon after starting sexual life and is mostly prevalent in young adults [8, 9]. A previous study suggests an estimated 6.2 million HPV infection annual incidence in people ranging from 14 to 44 years old [10]. Moreover, the overall estimated prevalence of HPV cases (genital warts) in Spain has been notified to be 118/100,000 inhabitants [11]. Several studies have shown that HPV infection has a negative psychosocial impact on well-being and health-related quality of life (HRQoL), both low-risk HPV inducing genital warts and high-risk HPV inducing (pre-) cancerous lesions [12–14]. Patients with HPV infection experienced heavier psychosocial burden than the general population, and females experienced higher burden than males [12–14]. HPV infection impact on HRQoL dimensions depending on the questionnaire used, but those related with self-image and sexual impact were usually highly impacted [15, 16]. Literature has also found other health areas with a substantial concern among people with HPV, including anxiety, stress, and detriments in sexual functioning [17–20]. Anxiety is significantly higher in women testing HPV-positive than those without HPV infection [21]. Participating in a routine screening can cause pain, embarrassment, fear, and inconvenience, even when the tests detect no abnormalities [20–22]. Despite the wide previous literature, few researchers have used specific questionnaires to measure the psychosocial effects of HPV-infection. Many studies have used generic instruments to measure HPV-infected women feelings such as anxiety or depression, but these tools are broad and may not capture all the salient features caused by the condition. Although some HPV-specific questionnaires have been described, few have been formally developed or validated. The HPV impact profile (HIP) was developed to assess the psychosocial impact of HPV infection and related interventions. The “Cuestionario Específico para Condiloma Acuminado” (CECA-specific questionnaire for condyloma acuminata) [23–25], only explores the impact of having genital warts, a particular presentation of HPV clinical spectrum, in patient quality of life. The Functional Assessment of Chronic Illness Therapy-Cervical Dysplasia (FACIT-CD) assesses the quality of life related to the finding of cervical dysplasia, focusing on the physical and psychological fields, but is tedious and should be validated in further studies [26]. To our knowledge, there is no comprehensive instrument designed to evaluate the full spectrum HPV infection-health related quality-of-life impact in women. The HPV-Quality-of-Life (HPV-QoL) questionnaire was developed to determine the impact of HPV infection and related interventions in women HR-QoL. This study provides, for the first time, the development of a new HPV-QoL questionnaire in adult women with HPV along with its preliminary psychometric properties.

## Materials and methods

### Panel of experts

The questionnaire development process began with the selection of a panel of eight experts composed of six gynaecologists specialized in lower genital tract diseases, one gynaecologist specialist in sexuality and HRQoL, and one methodologist expert in HRQoL. Every step of the development and validation of the questionnaire was supervised by the panel of experts. A literature review and compilation of the articles published about the impact of HPV and/or genital warms in different life aspects of woman HRQoL were first carried out. This search included Medline, Embase, Current Contents, Cochrane Library, and Google scholar. Taking these reviews as a starting point, the panel of experts generated an initial set of questions related to the impact of HPV on different domains of woman quality-of-life: social wellbeing, psychological wellbeing, and interference with activities of daily-living, mental health, concern about infecting other people, worrisome about general health consequences, anxiety, depression sensation, sexual desire, and characteristics of sexual relationship.

### Patient’s interviews

Patient’s interviews were carried out to record their opinion on the relevance of the dimensions and items initially considered by the panel of experts, and to collect additional information about those aspects of treatment the expert’s panel might have missed. Patients were questioned about the time during which they had been infected by HPV, their concern about the impact on daily-living, general health possible consequences and patient’s feelings, sensation, and usual behaviour after getting HPV. A total of 20 patients were interviewed in outpatient gynaecology clinic in the Community of Madrid and Málaga (Spain).

### Item generation

**Fig. 1:**
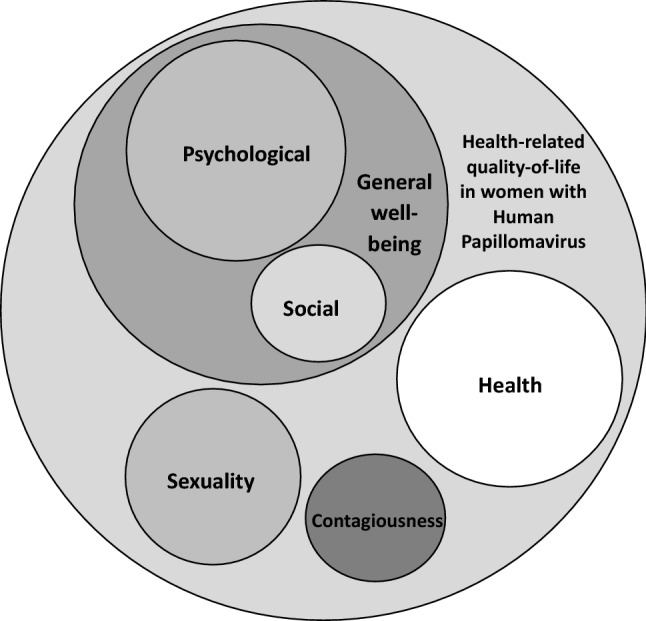
Conceptual modelling of the Human Papillomavirus-Quality of Life (HPV-QoL) questionnaire

Combining the initial considerations from the experts, literature review and the information recorded from the patient´s interviews, dimensions held to be relevant were included in the questionnaire, thus generating a comprehensive list of items in affirmative format and reflecting the expressions directly recorded from the patients interviews at its most. The items were designed to ensure they referred to a single concept, avoiding double negation and ambiguity, and using a first-person format. The answers were scored on a Likert-type scale from 1 to 5, as follows: 1 = “Totally agree”; 2 = “Agree”; 3 = “Neither agree nor disagree”; 4 = “Disagree” and 5 = “Totally disagree” [27]. The initially formulated items were evaluated by a semantic discussion and screening process, resulting in 20 items grouped into 4 domains (Fig. 1): General well-being including social well-being and psychological well-being (subdomains), sexuality, concern about infecting other people and worrisome about general health consequences. Social well-being focuses on patient interferences with activities of daily-living and psychological well-being evaluates different aspects related to woman emotional status. Sexuality domain assesses arousal, sexual desire and satisfaction with sexual relations.

### Subjects

Since the new instrument aims to measure the impact of HPV infection on women HRQoL, its development and validation were based on the selection of adult women previously diagnosed with HPV infection. For patient recruitment, the researchers conducted a systematic sampling in two hospital centres: Hospital Clínico San Carlos (Madrid) and Hospital Quirón (Málaga). Patients were selected if they met the following inclusion criteria: outpatient women over 18 years of age, with HPV infection diagnosis irrespective of previous vaccination against HPV, ability to understand and answer health questionnaires provided in Spanish language (Spain), and willing to sign the informed consent form. Exclusion criteria were pregnancy at the time of the assessment, recent diagnosis of any malignancies and current diagnosis of psychiatric disorders. A multicentre cross-sectional study was conducted under the conditions of usual clinical practice regarding disease treatment, and all patients were requested to grant informed consent.

Three different samples were used: (1) *pilot sample*: composed of 30 randomly recruited patients to check feasibility, pertinence and understandable items; (2) *reduced sample*: the first version of questionnaire was administered to a limited sample of 102 women (mean [SD] age: 38.0 [8.6] years, range: 21 to 65 years old) with HPV infection to confirm feasibility and to check eventual items redundancy, wording of items, rating system (5-categories Likert scale) and to explore the scale dimensionality [27]; (3) *validation sample*: this sample was formed by 252 women with HPV infection enrolled systematically from the Low Genital Tract Unit and fulfilling inclusion–exclusion criteria mentioned above, and was used to check psychometric properties of the HPV-QoL questionnaire. The size of the validation sample was determined based on the criterion of Rummel [28], whereby the ratio subjects/variables should be 4/1 to 7/1. Considering the number of items of the first version of the questionnaire, a minimum of 150 patients was considered an adequate sample. The size of the validation sample was over-dimensioned to allow statistical comparisons between meaningful groups related to the validity study (known group validity) and to explore initial composition of the norms of the scale. Thus, a minimum of 250 women were selected. Table 1 reports the main demographics data of the validation sample.

**Table 1:** Demographic and clinical data of women (*n* = 252) included in the psychometric validation study of the HPV-QoL questionnaire

Variable	Mean (SD) or %
Age (years), mean (SD)	38.9 (9.0)
BMI (kg/m^2^), mean (SD)	23.9 (4.2)
No Obesity (< 30)	91.4
Obesity (≥ 30)	8.6
Smoking (1 or more cigarettes per day) (%)	22.3
Daily alcohol consumption (%)	
No	28.7
Sometimes	68.9
Daily	2.4
Educational level (%)	
Primary	8.6
High school/LT	32.2
University	59.2
Gestations (%)	
0	54.1
1	18.3
2 +	27.6
Deliveries (%)	
0	58.9
1	19.0
2	16.9
3 +	5.2
Menopause status (%)	
No	81.9
Peri	6.0
Post	12.1
Sexual activity (%)	
No	14.1
Occasional	23.3
Regular	62.7
Immunosuppression (%)	
No	93.8
Yes	2.9
HIV	3.3
Active HPV infection (%)	
No	22.9
Yes	77.1
Previous STI (%)	
No	91.6
Yes	8.4
Contraception (%)	
No	26.9
Yes	73.1

SD standard deviation, BM body mass index, *LT* labor trainee, *HPV* human papilloma virus, *HIV* human immunodeficiency virus, *STI* sexually transmitted infection

### Reduction of the questionnaire

The initial 20 items of the questionnaire were administered to the pilot sample. Patient comments and information about comprehension and reading problems with the proposed items were collected. Contributions of the pilot sample were incorporated to the questionnaire and it was in turn administered to the reduction sample. Data obtained from this sample were then used for the following: (1) to check adjustment of the patient responses to the structure (dimensions or subscales) proposed by the group of experts; (2) to assess the metric properties of the items; and (3) to reword four items considered confusing or need further clarification by interviewers. Reduction of the questionnaire and determination of the underlying dimensions were carried out using a sequence of exploratory factor analyses and were based on the analysis of internal consistency. This factor analysis made use of principal components extraction method. Principal axes method was also explored. Rotation methods included varimax, direct oblimin, promax, quartimax and equamax [24]. The optimum number of factors was determined by application of the Kaiser K1 rule, the percentage of variance accounted for, and the magnitude of the eigenvalues after rotation [29–32]. Internal consistency was studied with the Cronbach’s alpha reliability coefficient and the change in alpha coefficient after deleting each item from the scale [29–32].

In the reduction of the length of the questionnaire and analysis of dimensionality, we adopted the proposals of Gorusch and Russell [33–35]. Firstly, we discarded those items with a clear floor or ceiling effect (i.e., items with more than 50% of answers concentrated in the first or last answer category). Secondly, an exploratory factor analysis was made with the items of the scale to determine the number of underlying factors or dimensions (subdomains). Lastly, dimensionality (factor analysis) and internal consistency (Cronbach’s alpha coefficient) of each subscale were analysed, assuming them as unidimensional. In this latter step, those items with lower loading in the first dimension or cross-loading in more than one dimension were discarded. Items with lowest contribution to the scale overall alpha coefficient were also dismissed. After each deletion, the same analyses were repeated until the unidimensional structure in each subscale was found to be stable, showing no improvement in the alpha coefficient. Finally, an exploratory factor analysis was carried out with all the optimized subscales to check that the structure remained stable. Throughout this process, the initial questionnaire was reduced first to a version of 11 items and, subsequently, during the reduction phase and factor analysis exploration, four items were split, increasing the total number up to 15. This final version was tested in the validation phase.

### Psychometric properties of the final version

The final version of the questionnaire was included in a case report form (see Spanish and English versions of questionnaire in supplementary material), together with information about the clinical impact of the infection in daily life, sociodemographic information, general questions regarding HPV infection and its vaccination, sexual status and related therapies. Spanish versions of the GHQ-12 questionnaire from Goldberg et al. [36] to explore mental functioning, psychological well-being and coping, along with the Female Sexual Function Index were also included [37, 38]. The data obtained from this sample were then used for the following: (1) to assess the metric properties of the questionnaire; and (2) to elaborate exploratory norms for the Spanish female population with HPV infection.

The following metric properties were studied: (1) *feasibility:* administration time, floor and ceiling effects, percentage of missing values in each item; (2) *reliability*: internal consistency, evaluated by means of Cronbach’s alpha coefficient and the Pearson correlation coefficient among items and between each item and the total composite score. A test–retest was also performed to check the temporal stability. This was tested by asking 30 women to complete HPV-QoL questionnaire for a second time at a mean of 1.6 days (range 1–3 days) after first administration and evaluated using an intraclass correlation coefficient (ICC) with a mixed effect two factor model and total agreement [39–41]; (3) *content validity*: ensured by the panel of experts and (4) *construct validity*: was tested by means of different approaches, convergent and divergent analysis was carried out by estimating the Pearson moment r coefficient between each domain, between domain and items and between each item to analyse correlations between items and domains of the questionnaire. Extrinsic construct validity was explored by means of estimating the *concurrent validity* of the HPV-QoL domains and total scores with those of the GHQ-12 and the Female Sexual Function Index. This estimation was performed by calculating Pearson r coefficients. *Known groups validity* was performed to analyse the power of the HPV-QoL total and domain scores to discriminate between groups of women with different responses in clinical and demographic criteria that were hypothesized a priori to be different. These criteria included the split of the sample in two subgroups according to responses in GHQ-12 (mental deterioration; GHQ-12 total score < or ≥ 17 points) and sexual dysfunction (FSFI scale > or ≤ 26.55 points) [38, 42]. Differences were analysed with a general linear model including covariates (age, body mass index, menopause, antidepressant therapy, sexual activity, and STIs). Bonferroni adjustment was performed in case of multiple comparisons and estimation of the 95% confidence interval of differences by non-parametric 1000 bootstrap iterations. Magnitude of differences were calculated by determining the statistic effect size [43, 44]. All analyses were made using the IBM SPSS version 20.0, NY, USA, statistical package (https://www.ibm.com/analytics/spss-statistics-software).

### Scoring

Summing up the direct scores of the items yields a total composite score ranged from 15 to 75. This total composite score can be transformed to a more intuitive and easier to understand metric with a minimum of 0 (worst quality-of-life) and a maximum of 100 (best quality-of-life), using the following expression: *Y′* = [*Y*_*obs*_ − *Y*_*min*_] / [*Y*_*max*_ − *Y*_*min*_] × 100  = *Y*_*obs*_; in which *Y*_max_ = 75 (maximum total score); *Y*_min_ = 15 (minimum total score); *Y*_obs_ = total score obtained by the patient; and *Y′* = transformed score. A similar expression can be used to change the metric of each individual dimension. To facilitate interpretation, a decile distribution was implemented for the total score questionnaire and dimensions as a proxy of scale norms.

### Reporting guidelines

The Strengthening the Reporting of Observational Studies in Epidemiology (STROBE) statement guidelines for reporting observational studies were followed www.strobe-statement.org.

## Results

The HPV-QoL questionnaire was administered to 252 women, 21 to 65 years of age, in which 18 (7.14%) left 26 of 3,780 items blank (0.69%). The mean completion time was 4.99 (DE = 3.92) minutes (range: 1 to 15 min). Completion time was not statistically associated with either educational level (*F* = 0.97, *p* = 0.382) or age (*r* = − 0.032, *p* = 0.638). The proportion of unanswered items was not statistically significant regarding the level of studies (linear Chi2 = 2.354, *p* = 0.125) or age (*t* = 0.325, *p* = 0.745). No correlation between age and total scores or scale dimensions was found as rho coefficients ranged from − 0.051 to 0.089 (*p* = 0.159 or greater). Table 1 shows the main demographic and clinical data.

### Factorial structure and components

Taking the Kaiser criterion of eigenvalues equal to or greater than 1 and the visual examination of the Cattel sedimentation graph as a reference, the results obtained using factor analysis revealed the presence of a multifactorial solution with four dimensions (Table 2), which explains 69.37% of the total variance (determinant of the matrix = 0.000; KMO = 0.88; Bartlett’s sphericity test; Chi2 = 2040.813, *p* < 0.0001). The non-additive test showed an F-statistic of 9.827 (*p* = 0.002) ruling out the additive hypothesis of the questionnaire. In all items, high factorial weights were observed in their corresponding dimensions, ranging from 0.638 of item 12 to 0.903 of items 14 and 15.

**Table 2:** Principal component analysis supporting a factorial structure with four domains confirming the conceptual model of the questionnaire HPV-QoL

Component/domain	Total	Extraction		Rotation solution		
		Variance (%)	Cumulated (%)	Total	Variance (%)	Cumulated (%)
1	6.247	41.645	41.645	3.506	23.372	23.372
2	1.945	12.969	54.614	3.278	21.852	45.224
3	1.254	8.362	62.975	2.341	15.605	60.829
4	0.959	6.395	**69.370**	1.281	8.541	**69.370**

Item	Components			
	1 General well-being	2 Health	3 Sexuality	4 Contagiousness
e1	**0.648**	0.582	0.207	0.332
e2	**0.852**	0.540	0.440	0.407
e3	**0.831**	0.541	0.351	0.407
e4	**0.693**	0.669	0.471	0.459
e5	**0.832**	0.232	0.474	0.162
e6	**0.832**	0.156	0.473	0.179
e7	0.151	0.314	0.130	**0.826**
e8	0.423	0.188	0.205	**0.672**
e9	0.253	**0.800**	0.168	0.371
e10	0.322	**0.856**	0.236	0.249
e11	0.371	**0.847**	0.273	0.269
e12	0.456	**0.638**	0.424	0.238
e13	0.658	0.518	**0.770**	0.277
e14	0.440	0.266	**0.903**	0.166
e15	0.438	0.275	**0.903**	0.210
Auto engines	**6.25**	**1.95**	**1.25**	**0.96**

Principal component analysis. Rotation method: Promax normalization with Kaiser. In bold highest saturation coefficients

The first factor (1) was “*General well-being*”. It included items 1 to 6 and explained 41.65% of the total variance and showed two sub domains that explained 79.74% of the variance of this factor and that were called “*Psychological well-being*” (items 1 to 4) and “*Social well-being*” (items 5 and 6). The second factor, which explains 12.97% of the variance, has been labelled as “*Health*” and contains items 9 to 12. The third factor explains 8.36% and has been labelled as “*Sexuality*” (items 13 to 15) and the fourth factor explains 6.40% and was labelled as “*Contagiousness*” (items 7 and 8).

### Reliability and variability

Table 3 shows reliability and variability of the HPV-QoL questionnaire together with the mean (SD) scores in each domain and the total questionnaire. Missing items were negligible (less than 2%) and floor and ceiling effects were acceptable except for a ceiling effect of 100% in social well-being subdomain. Internal consistency coefficient was above 0.7 in total score and in every domain apart from contagiousness, with a lower Cronbach´α coefficient (0.285). Temporal stability was supported by a test–retest ICC punctuation above 0.7 in total score (0.817) and in every domain, except contagiousness, with a moderate score of 0.533.

**Table 3:** Reliability and variability of the HPV-QoL questionnaire in the validation sample (*n* = 252) of adult Spanish women

Domain (# items)	Score			Cronbach-α	Test–retest ICC	SEM	Items missed (%)	Floor effect (%)	Ceiling effect (%)
	Mean	SD	Range (high-low)						
General well-being (6)	58.52	24.22	0–100	0.881	0.871	8.36	0.26	0.0	33.3
Psychological (4)	47.05	27.94	0–100	0.871	0.832	10.04	0.20	0.0	0.0
Social (2)	81.45	24.20	0–100	0.902	0.859	7.58	0.40	0.0	100.0
Health (4)	23.64	21.86	0–88	0.807	0.692	9.60	1.98	50.0	0.0
Contagiousness (2)	46.23	24.00	0–100	0.285	0.533	20.21	0.10	0.0	0.0
Sexuality (3)	62.96	29.46	0–100	0.836	0.729	11.93	1.46	0.0	0.0
Total (15)	47.84	18.76	0–88	0.894	0.817	6.11	0.69	13.3	13.30

*SD* standard deviation, *ICCI* intraclass coefficient of correlation, *SEM* standard error of measurement; Floor and ceiling effects: % of items in each domain with 50% or above of respondents in the highest or lowest category of response, respectively

### Construct validity

**Fig. 2:**
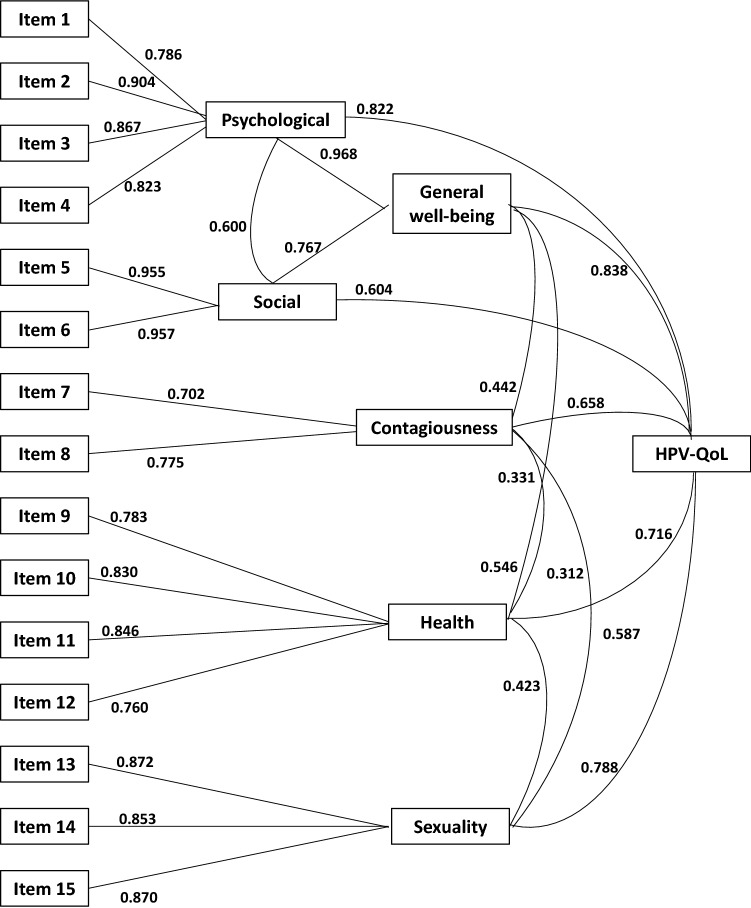
Construct validity of the HPV-QoL questionnaire: convergent and divergent validity total to domain, domain to domain and domain to item

Intrinsic convergent and divergent validity is shown in Table 4 and Fig. 2. Table 4 includes correlation coefficients of convergent and divergent validity for item to item and item to domain. Figure 2 includes convergent and divergent validity for total score to domains, domain to domain and domain to items. Most of the correlation coefficients showed higher values, with items in its own domain or total domain, than with items in other domains of the scale. Likewise, items correlations with total score were lower than with items in its own domain. Extrinsic construct validity was supported by means of exploring concurrent validity of the HPV-QoL questionnaire (total score and domains) with FSFI and GHQ-12 questionnaires in total score and domain scores. Table 5 includes correlation coefficients of HPV-QoL with FSFI and GHQ-12, showing highest values of moderate intensity in domains that were related at first: Sexuality in HPV-QoL showed good correlation with all domains and total score of the FSFI. Well-being (general, psychological, and social) had also strong correlation with all domains and total score of GHQ-12. Additionally, total scores of HPV-QoL and GHQ-12 showed correlation coefficients with moderate intensity.

**Table 4:** Construct validity of the HPV-QoL; convergent and divergent validity item to item and item to dimension

Item	e1	e2	e3	e4	e5	e6	e7	e8	e9	e10	e11	e12	e13	e14	e15
e1	1.00														
e2	0.605	1.00													
e3	0.564	0.745	1.00												
e4	0.560	0.667	0.623	1.00											
e5	0.311	0.609	0.582	0.422	1.00										
e6	0.326	0.589	0.583	0.435	0.844	1.00									
e7	0.254	0.234	0.234	0.354	**0.063**	**0.057**	1.00								
e8	0.195	0.363	0.368	0.280	0.351	0.365	0.151	1.00							
e9	0.360	0.412	0.383	0.406	0.206	0.138	0.305	0.186	1.00						
e10	0.417	0.383	0.385	0.458	0.202	0.144	0.301	0.131	0.696	1.00					
e11	0.370	0.420	0.442	0.486	0.262	0.187	0.287	0.164	0.643	0.738	1.00				
e12	0.412	0.395	0.420	0.512	0.305	0.301	0.264	0.144	0.393	0.435	0.480	1.00			
e13	0.425	0.580	0.492	0.598	0.488	0.525	0.234	0.266	0.331	0.365	0.382	0.552	1.00		
e14	0.194	0.398	0.330	0.372	0.392	0.417	**0.060**	0.316	0.168	0.168	0.204	0.272	0.591	1.00	
e15	0.192	0.400	0.307	0.384	0.362	0.397	0.118	0.277	0.189	0.161	0.193	0.285	0.587	0.755	1.000

Item/domain	e1	e2	e3	e4	e5	e6	e7	e8	e9	e10	e11	e12	e13	e14	e15
General well-being	0.719	0.900	0.867	0.781	0.735	0.739	0.262	0.402	0.412	0.432	0.471	0.496	0.649	0.431	0.418
Psychological	0.786	0.904	0.867	0.823	0.575	0.578	0.305	0.360	0.459	0.477	0.503	0.494	0.609	0.380	0.375
Social	0.332	0.620	0.607	0.447	0.955	0.957	**0.061**	0.368	0.174	0.169	0.225	0.318	0.531	0.417	0.380
Contagiousness	0.294	0.391	0.407	0.415	0.277	0.292	0.702	0.775	0.317	0.261	0.281	0.251	0.315	0.244	0.238
Health	0.460	0.472	0.493	0.567	0.304	0.248	0.334	0.198	0.783	0.830	0.846	0.760	0.516	0.263	0.264
Sexuality	0.331	0.536	0.444	0.538	0.471	0.507	0.177	0.318	0.287	0.288	0.314	0.449	0.872	0.853	0.870
Total-HPV-QoL	0.576	0.763	0.717	0.744	0.587	0.581	0.459	0.548	0.563	0.569	0.596	0.613	0.784	0.617	0.617

Item to item convergent and divergent validity (correlation coefficient)

Item to domain convergent and divergent validity

Sperman´s rho coefficients; in bold correlation coefficients not significant at *p* < 0.05 level. Rest of coefficients significant at *p* < 0.05

**Table 5:** Construct validity: concurrent validity of HPV-QoL questionnaire with FSFI and GHQ-12 questionnaires

	HPV-QoL questionnaire						
	General well-being	Psychological well-being	Social well-being	Contagiousness	Health	Sexuality	Total score
FSFI questionnaire							
Desire	0.203	0.17	0.212	**0.085**	**0.08**	0.466	0.301
Arousal	**0.129**	**0.102**	0.149	− **0.002**	**0.108**	0.346	0.209
Lubrication	**0.119**	**0.093**	0.138	**0.005**	**0.091**	0.31	0.189
Orgasm	**0.086**	**0.067**	**0.101**	**0.012**	**0.128**	0.279	0.179
Satisfaction	**0.127**	**0.116**	**0.112**	**0.034**	**0.085**	0.292	0.192
Pain	**0.087**	**0.071**	**0.095**	− **0.023**	**0.067**	0.335	0.172
Total score	**0.135**	**0.111**	0.146	**0.015**	**0.106**	0.373	0.226
GHQ-12 questionnaire							
Self-esteem	− 0.521	− 0.488	− 0.433	− 0.251	− 0.338	− 0.393	− 0.502
Stress	− 0.515	− 0.501	− 0.388	− 0.327	− 0.411	− 0.337	− 0.523
Coping	− 0.336	− 0.312	− 0.286	− 0.219	− 0.153	− 0.256	− 0.324
Total score	− 0.56	− 0.532	− 0.451	− 0.313	− 0.38	− 0.403	− 0.551

In bold correlation coefficient not significant at *p* < 0.05 level. Rest of coefficients significant at *p* < 0.05

*FSFI* female sexual function index (Rosen et al.), *GHQ-12* general health questionnaire (Golberg et al.)

**Fig. 3:**
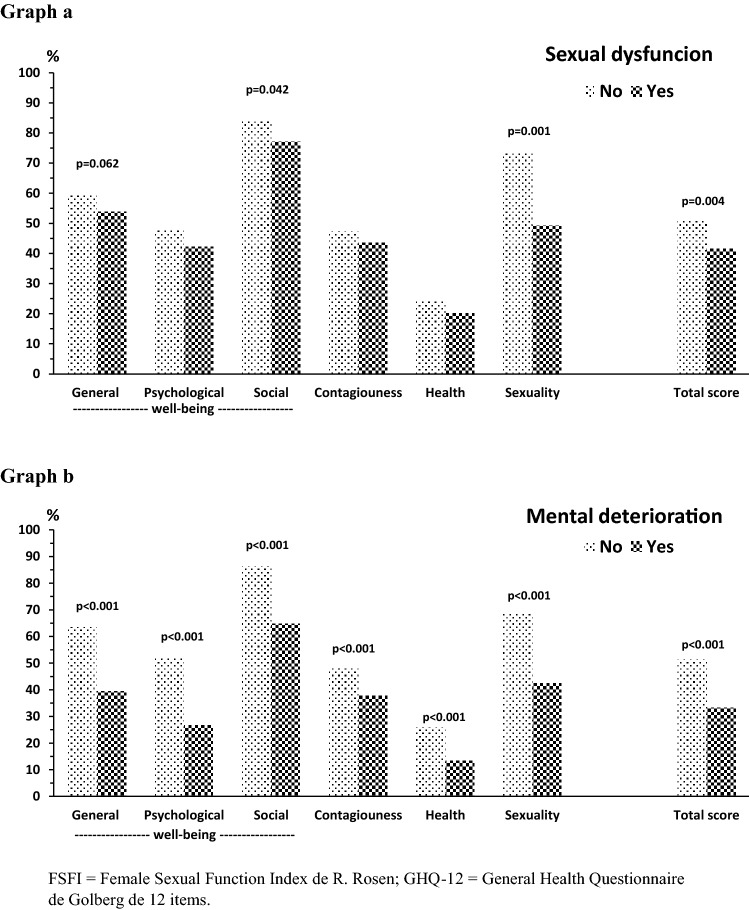
Known groups validity of HPV-QoL questionnaire in women according to presence of sexual dysfunction (score in questionnaire FSFI ≤ 26.55 points, graph **a**) or mental deterioration by GHQ-12 questionnaire (≥ 17 points, graph **b**). FSFI = Female Sexual Function Index de R. Rosen; GHQ-12 = General Health Questionnaire de Golberg de 12 items

Finally, construct validity was supported by discriminant or known groups validity. Figure 3 shows that domain scores and total score of HPV-QoL were significantly different according to the presence of sexual dysfunction. Significantly lower punctuation in total score, sexuality and general well-being in women with sexual dysfunction than those without sexual dysfunction was found. Also, HPV-QoL total and all domain scores were significantly lower in women with mental deterioration according with GHQ-12 than those without mental deterioration (Fig. 3). Magnitude of differences as assessed by effect size calculation were moderate to high (> 0.40) in all domains when GHQ-12 was applied and in sexuality and total score when FSFI was used (Table 6). Table 7 shows preliminary normative values for HPV-QoL questionnaire.

**Table 6:** Known groups validity effect sizes for active HPV infection, HPV vaccination, sexual activity, menopause status, antidepressant therapy and sexually transmitted infection

	Effect size					
	Active HPV	HPV vaccination	Sexual activity	Menopause	Sexually transmitted infection	Antidepressant therapy
Wellbeing					0.46	
Psychologic					0.48	0.30
Social						
Contagiousness						
Health						
Sexuality	0.40		0.60	0.49		
Total-HPV score	0.34		0.46	0.21		0.30

**Table 7:** Interpretability of the HPV-QoL questionnaire: decil values by domain and total score in the validated sample of 252 women 21–65 years old

Percentile	Domain						Total score
	General well-being	Psychological well-being	Social well-being	Contagiousness	Health	Sexuality	
10	25.00	12.50	50.00	0.00	0.00	16.67	23.07
20	37.50	25.00	62.50	25.00	0.00	33.33	32.29
30	45.83	31.25	75.00	37.50	6.25	50.00	36.46
40	50.00	37.50	75.00	50.00	12.50	58.33	43.33
50	58.33	43.75	100.00	50.00	18.75	66.67	47.40
60	66.67	56.25	100.00	50.00	25.00	75.00	51.56
70	75.00	62.50	100.00	62.50	31.25	83.33	56.87
80	79.17	68.75	100.00	62.50	43.75	91.67	65.63
90	91.67	87.50	100.00	75.00	56.25	100.00	73.85

## Discussion

The main goal of the female Lower Tract Disease Units is to improve the accuracy in the diagnosis of the HPV-related diseases. This aim has been traditionally focused on the evolution of the terminology, teaching and colposcopy technologies [45–47]. Through the last decades, the patient feelings have become more relevant when assessing their health status. In women with HPV infection, quality of life evaluation may constitute an essential point in decision-making. This study delves into the development of the metric properties of a new specific instrument to measure HRQoL in HPV infected women. According to the results, this clinical tool, called HPV-QoL questionnaire, in its paper and pencil version, is valid, reliable and feasible to use in the daily clinical practice. It can be implemented as a unidimensional instrument, if used as a total composite score.

The results reveal that the HPV-QoL questionnaire provides very good metric properties. Regarding its *feasibility*, the response rate is highly satisfactory, as almost all patients answered every question with negligible missing items, and irrespective of age and educational level. Additionally, time to administer the instrument was very brief: less than 5 min on average—thus making it feasible to use at any level of healthcare and particularly among outpatients, whose time for the visit is usually short. The *reliability* of the questionnaire, internal consistency, and temporal stability showed values above the acceptable standards [48, 49], as related to the total composite score and in terms of the individual domains, except for dimension contagiousness, in which internal consistency coefficient was low. It should be noticed that social well-being subdomain was significantly associated with ceiling effect. The different aspects analysed in relation to the *validity* of the questionnaire have also led to satisfactory results. The study of the responses based on exploratory factor analysis, and the relationship between items, items to domains and total score, supported the initially proposed theoretical structure. Specifically, the present study corroborated the presence of four main domains, with two subdomains: general well-being (this with subdomains psychological well-being and social well-being), health, contagiousness, and sexuality. The combination of all domains provides a composite meaningful total score that represents the impact of the HPV infection on women HRQoL. The instrument was able to correlate with domains of GHQ-12, including stress, self-esteem, coping and total mental score. In addition, total and sexuality domain scores of the HPV-QoL questionnaire were significantly correlated (with moderate correlation coefficient) with all domains of FSFI questionnaire as hypothesized initially. Finally, this study stresses the ability of the questionnaire, based on the total score and domains, to discriminate among groups that were assumed to be different because of the presence of either sexual dysfunction or mental deterioration. This last statement is another important component of construct validity testing. Total and sexuality domain scores were significantly different in presence of sexual dysfunction, showing moderate to high magnitude effect sizes. Furthermore, total and specific domain scores were able to find significant differences among women with mental deterioration assessed with GHQ-12, with moderate to high effect sizes. Likewise, given the limited number of participants used to test instrument properties, the present study was able to identify preliminary norms of the tool that should be confirmed with larger samples.

The HPV-QoL questionnaire offers several advantages over another existing related instrument [23–26]. Although some HPV-specific questionnaires have been described, few have been formally developed or validated. According to the previous literature, few questionnaires can evaluate the psychosocial impact of abnormal Papanicolaou tests or cervical disease [26, 50–54]. Moreover, this is the first study that displays a scale that focus on the impact of HPV-infection in women concerning their perception and feelings. The CECA questionnaire is an instrument only validated for women with genital warts [24, 25], being able to contrast the quality of life in patients with these genital lesions compared with the general population [25, 55]. The HPV Impact Profile (HIP) was developed as a self-administered tool to assess the psychosocial impact of an abnormal Papanicolaou test, CIN of any grade severity, and genital warts. Nonetheless, it does not analyse the total spectra of HPV infection impact [23].

The present study has as a limitation its cross-sectional design that restricts the possibility to examine longitudinal properties of the instrument such as sensitivity to change, responsiveness or predictive validity. Further studies will be needed regarding the ability of the instrument to detect changes in women health status, patient response to treatment or has a role in anticipating prognosis of HPV infection.

## Conclusion

The HPV-QoL questionnaire is a novel patient-reported outcomes specific measure to assess health-related quality-of-life in women with HPV infection. The findings of the study suggest that the instrument has good acceptability as well as satisfactory psychometric properties, including validity and reliability of the composite total score. The findings support the use of the HPV-QoL questionnaire as a HRQoL measurement tool in daily medical practice and clinical research.

## Appendix 1

### HPV-QoL questionnaire in original Spanish for Spain and English for United Kingdom

#### Cuestionario de Calidad de Vida Relacionada con la Salud HPV-QoL

Si padece una infección por el virus del papiloma humano (VPH), por favor lea atentamente cada una de las preguntas que vienen a continuación. Le preguntaremos sobre sus sentimientos y opiniones sobre aspectos relacionados con el VPH en el momento actual. En cada pregunta, marque con una X el número con el que considere estar más de acuerdo o en desacuerdo. No piense demasiado las respuestas, no hay respuestas buenas o malas, todas deben responderse con sinceridad. Quizá considere preguntas demasiado personales; no se preocupe, el cuestionario es totalmente anónimo y confidencial.

**Recuerde contestar todas las preguntas**. Si hay una pregunta en blanco el cuestionario se podría invalidar.

**Table:**

En el momento actual	Totalmente de acuerdo	De acuerdo	Ni de acuerdo ni en desacuerdo	En desacuerdo	En total desacuerdo
1. Pienso que tener el VPH me ha cambiado la vida	1	2	3	4	5
2. Me siento deprimida desde que sé que tengo el VPH	1	2	3	4	5
3. Estoy nerviosa desde que sé que tengo el VPH	1	2	3	4	5
4. Me siento más insegura teniendo el VPH	1	2	3	4	5
5. Saber que tengo el VPH interfiere con mis actividades cotidianas (tareas de casa, trabajo, estudiar, etc.)	1	2	3	4	5
6. Saber que tengo el VPH interfiere con mis actividades sociales (fiestas, quedar con amigos, tiempo libre, etc.)	1	2	3	4	5
7. Me preocupa contagiar el VPH a mi pareja	1	2	3	4	5
8. Tengo miedo de contagiar el VPH a los que me rodean (hijos, familiares, amigos)	1	2	3	4	5
9. Estoy preocupada por si el VPH me provoca un cáncer	1	2	3	4	5
10. Tengo miedo de que no desaparezca el VPH	1	2	3	4	5
11. Estoy ansiosa de saber si me voy a recuperar bien de las lesiones que produzca el VPH	1	2	3	4	5
12. No me fio al tener relaciones sexuales por miedo a contagiarme	1	2	3	4	5
13. Desde que sé que tengo el VPH se ha reducido mi deseo de tener actividad sexual (coito, caricias, masturbación)	1	2	3	4	5
14. Desde que sé que tengo el VPH me molestan las relaciones sexuales	1	2	3	4	5
15. Desde que sé que tengo el VPH mis relaciones sexuales son menos placenteras	1	2	3	4	5

*Corrección*: la puntuación total y en dimensiones oscila entre 0 y 100, siendo 0 la peor y 100 la mejor calidad de vida. Si una pregunta no se responde, la puntuación de la pregunta no contestada es imputada con la respuesta más frecuente observada en los otros ítems de la misma dimensión.

**Dimensiones**

**Table:**

•Bienestar general = [(P1 + P2 + P3 + P4 + P5 + P6)–6]/24 *100 oPsicológico = [(P1 + P2 + P3 + P4)–4]/16 *100 oSocial = [(P5 + P6)–2]/8 *100	•Salud = [(P9 + P10 + P11 + P12)–4]/16*100
•Contagio = [(P7 + P8)–2]/8 *100	•Sexualidad = [(P13 + P14 + P15)–3]/12 *100
Puntuación total = [Bienestar general + Contagio + Salud + Sexualidad]/4	

## HPV health-related quality of life questionnaire-HPV QoL

In you have human papillomavirus infection (HPV), please carefully read each of the following questions. You will be asked about your feelings and opinions related to HPV infection. For each question, please mark with a cross the number with which you most agree or disagree. Do not overthink the questions, there are not right or wrong answers; every question must be answered truthfully. You might consider some questions as too personal; do not worry, this questionnaire is fully anonymous and confidential.

**Please answer every question**. If you leave a question blank, questionnaire might become invalid.

**Table:**

At the present time	Totally agree	Agree	Nor agree or disagree	Disagree	Totally disagree
1. I think having HPV infection has changed my life	1	2	3	4	5
2. I feel depressed since I know I have HPV infection	1	2	3	4	5
3. I feel nervous since I know I have HPV infection	1	2	3	4	5
4. I feel insecure since I know I have HPV infection	1	2	3	4	5
5. Knowing I have HPV infection interferes with my daily activities (housekeeping, working, studying, etc.)	1	2	3	4	5
6. Knowing I have HPV infection interferes with my social activities (partying, meeting friends, working, studying, leisure time etc.)	1	2	3	4	5
7. I feel worried about infecting my partner	1	2	3	4	5
8. I feel worried about infecting my partner those around me (children, family, friends)	1	2	3	4	5
9. I feel worried about if HPV can cause me cancer	1	2	3	4	5
10. I feel worried about if HPV does not disappear	1	2	3	4	5
11. I feel anxious to know if I could recover from HPV related lesions	1	2	3	4	5
12. I do not feel confident when having sexual intercourse because of the risk of infection	1	2	3	4	5
13. Since I know I have HPV infection my sexual desire has decreased (intercourse, affection, masturbation)	1	2	3	4	5
14. Since I know I have HPV infection; sexual intercourse bothers me	1	2	3	4	5
15. Since I know I have HPV infection; sexual intercourse is a less delightful experience	1	2	3	4	5

*Clarification*: Overall score and dimension score ranges from 0 to 100, being 0 the worst score and 100 the best. If a question remains unanswered, the question score will be considered as the most frequently answered score in the dimension to which the item belongs.

**Dimensions**

**Table:**

•General well-being = [(P1 + P2 + P3 + P4 + P5 + P6)–6]/24 *100 oPsychological = [(P1 + P2 + P3 + P4)–4]/16 *100 oSocial = [(P5 + P6)–2]/8 *100	•Health = [(P9 + P10 + P11 + P12)–4]/16 *100
•Contagiousness = [(P7 + P8)–2]/8 *100	•Sexuality = [(P13 + P14 + P15)–3]/12 *100
Overall score = [General well-being + Contagiousness + Health + Sexuality]/4	
